# Prescribed Burning Enhances the Stability of Soil Bacterial Co-Occurrence Networks in *Pinus yunnanensis* Forests in Central Yunnan Province, China

**DOI:** 10.3390/microorganisms13092070

**Published:** 2025-09-05

**Authors:** Yunxian Mo, Xiangwei Bu, Wen Chen, Jinmei Xing, Qiuhua Wang, Yali Song

**Affiliations:** 1 College of Ecology and Environment, Southwest Forestry University, Kunming 650224, China; myxappec123@swfu.edu.cn; 2 College of Soil and Water Conservation, Southwest Forestry University, Kunming 650224, China; 13583478644@163.com (X.B.); chenwen0610@163.com (W.C.); xingjinmei1720@163.com (J.X.); 3 College of Forestry, Sichuan Agricultural University, Chengdu 611130, China; 4 College of Civil Engineering, Southwest Forestry University, Kunming 650224, China; qhwang2010@swfu.edu.cn

**Keywords:** prescribed burning, soil microorganisms, soil physicochemical properties, co-occurrence network, key microorganisms

## Abstract

Prescribed burning significantly influences the microbial communities and physicochemical characteristics of forest soils. However, studies on the impacts of prescribed burning on the stability of soil microbial co-occurrence networks, as well as on the combined effects of post-fire soil depth gradients and their interactions on soil physicochemical properties and microbial communities, remain poorly understood. This study was conducted in a subtropical *Pinus yunnanensis* plantation that has undergone annual prescribed burns since 2007. Using 16S and ITS rRNA gene sequencing techniques alongside analyses of soil physicochemical properties, we collected and examined soil samples from different depths (0–5 cm, 5–10 cm, and 10–20 cm) in June 2024. The study found that prescribed burning enhanced the complexity and stability of bacterial co-occurrence networks, boosting both the diversity (prescribed burning/unburned control: 3/1) and the abundance (prescribed burning/unburned control: 8/2) of key taxa, which were essential for maintaining bacterial community network stability. However, it also intensified competitive interactions (prescribed burning/unburned control: 0.3162/0.0262) within the community. Moreover, prescribed burning had a significant effect on the diversity, structure, and composition of microbial communities and the physicochemical properties in the 0–5 cm soil layer, while also showing notable effects in the 5–20 cm layer. Prescribed burning also enhanced the coupling between the soil environment and bacterial community composition. The bacterial community showed negative correlations with most physicochemical properties. Soil organic matter (SOM) (*p* = 0.002) and available potassium (AK) (*p* = 0.042) were identified as key determinants shaping the post-fire bacterial community structure. The relationship between physicochemical parameters and fungal community composition was weaker. Urease (UE) (*p* = 0.036) and total potassium (TK) (*p* = 0.001) emerged as two key factors influencing the composition of post-fire fungal communities. These results elucidate the distinct functional roles of bacteria and fungi in post-fire ecosystem recovery, emphasizing their contributions to maintaining the stability and functionality of microbial communities. The study provides valuable insights for refining prescribed burning management strategies to promote sustainable forest ecosystem recovery.

## 1. Introduction

For over 350 million years, fire has acted as an important ecological driver, continuously reshaping terrestrial ecosystems, particularly since the emergence of plants [1,2]. It has become an essential component of most forest ecosystems, shaping forest dynamics and contributing to the complex biotic structures observed in contemporary landscapes [3]. Prescribed burning is a widely adopted forest management tool, especially in regions susceptible to wildfires, where it is employed to regulate surface fuel loads, diminish understory flammability, and reduce the likelihood and scale of wildfires [4,5]. Despite its widespread use, significant debate persists regarding the effects of prescribed burning on forest ecosystem functioning, particularly with respect to microbial communities, nutrient cycling, and soil microclimates [6,7].

Post-fire alterations in soil physicochemical properties and microbial community composition are critical indicators of soil recovery [8]. With their exceptionally high biodiversity and taxonomic richness, soil microorganisms exhibit faster and stronger responses to fire disturbances than macroorganisms, thereby being recognized as critical drivers of soil recovery following fire [9,10]. Surface soil temperatures during prescribed burns can reach extremes of 386–578 °C [11], far surpassing the thermal thresholds for most bacteria and fungi [12], leading to substantial microbial mortality. Furthermore, this thermal effect may be further amplified by reduced vegetation cover and soil moisture evaporation, leading to elevated soil temperatures that consequently affect subsurface microbial communities. Numerous studies also have shown that fire-induced changes in microbial communities occur at specific post-fire intervals and within particular soil depths (e.g., 0–2.5 cm, 0–5 cm, 5–10 cm, 0–10 cm, and 0–20 cm) [8,13,14,15], with the intensity of impact diminishing with increasing depth [13,16]. Moreover, the relationship between soil physicochemical properties and microbial community structure and diversity is inherently complex. Long-term prescribed burning can alter microbial community distribution by influencing soil redox potential and physicochemical gradients [8,17]. However, the majority of studies have focused on a single soil depth, and the effects of prescribed burning across multiple layers remain underexplored. Therefore, it is imperative to explore the impacts of prescribed burning on microbial communities across varying soil depths, especially considering soil microorganisms that can rapidly respond to environmental changes. These microbes can serve as critical bioindicators for predicting post-fire soil functional recovery and informing adaptive ecosystem management. Such investigations are essential for deepening our understanding of the impacts of prescribed burning on microbial communities and their associated ecological processes.

Microbial co-occurrence networks, which reveal the intricate relationships within microbial communities, are increasingly used to investigate microbial responses to environmental disturbances [18,19]. The stability of microbial co-occurrence networks is defined as the capacity of species interaction networks within microbial communities to maintain their structural integrity and functional performance under environmental fluctuations, external disturbances, or the loss of keystone taxa (resistance), as well as their ability to recover to the original state once disturbances are removed (resilience) [20]. Such stability provides a crucial basis for understanding microbial adaptability and ecological robustness following prescribed burning. Highly stable networks sustain microbial diversity and promote long-term community persistence, whereas unstable networks may drive structural simplification and increased modularity, thereby disrupting mutualistic and competitive interactions and weakening the community’s capacity for self-repair and resistance to perturbations [19]. While the effects of forest fires on microbial co-occurrence networks and stability have been well studied [18,21], research on prescribed burning is relatively limited. Keystone microbes play pivotal roles in maintaining both community functionality and network stability [20]. Under environmental disturbances, such key taxa can dominate, thereby exerting substantial influence over ecosystem processes [22]. Although numerous studies have investigated the role of keystone microorganisms in maintaining microbial community function and network stability following environmental disturbances [20,23,24], most have focused on general disturbance scenarios. In contrast, research remains limited regarding the dynamic changes in the abundance and diversity of keystone taxa under fire disturbance, as well as the specific mechanisms by which these taxa contribute to the stability of microbial co-occurrence networks. Therefore, further research is urgently needed in this field to elucidate the specific mechanisms by which keystone microorganisms contribute to post-fire ecosystem recovery.

In addition to fire severity, several factors- including burn frequency, seasonality, climate, vegetation composition, initial soil conditions, and overall ecosystem characteristics- interact to govern the post-burn recovery of soil microbial communities [8,25,26,27,28]. These factors interact in complex, nonlinear ways and display significant spatiotemporal variation. Although research on soil microbial recovery following prescribed burning has been conducted in northern China, the United States, and other regions, studies in tropical and subtropical forests, especially in fire-prone southwestern China, have primarily concentrated on fire prevention, risk assessment, and vegetation regeneration [11,17,29,30], with relatively few studies investigating the recovery of soil microbial communities. Investigating microbial community recovery following prescribed burning in southwestern China is therefore essential for advancing our understanding of microbial dynamics in various ecological contexts.

In light of the previously mentioned research advancements, this study aims to: (a) assess the variations in the structure, diversity, and composition of bacterial and fungal communities across different soil layers following prescribed burning; (b) identify the primary soil physicochemical factors influencing microbial community composition; and (c) investigate how prescribed burning affects key microbial taxa and co-occurrence networks. We hypothesized that: (1) prescribed burning would significantly alter the microbial community structure, diversity, and network dynamics, with soil depth influencing these changes; (2) soil physicochemical properties would shape microbial community composition, with distinct factors regulating communities in burned and unburned areas; (3) bacteria and fungi would exhibit differential responses to environmental disturbances; and (4) key microbial taxa would be essential for maintaining network stability. To test these hypotheses, the study was conducted in a *Pinus yunnanensis* forest at Zhaobi Mountain, Yuxi City, Yunnan Province, southwestern China. We used 16S and ITS rRNA gene sequencing to analyze microbial communities and evaluated physicochemical properties across different soil layers over a 16-month recovery period post-fire. This research offers novel insights into microbial community shifts following prescribed burning and introduces microbial network analysis as an innovative approach to evaluating post-fire ecosystem health.

## 2. Materials and Methods

### 2.1. Study Area and Design

**Figure 1:**
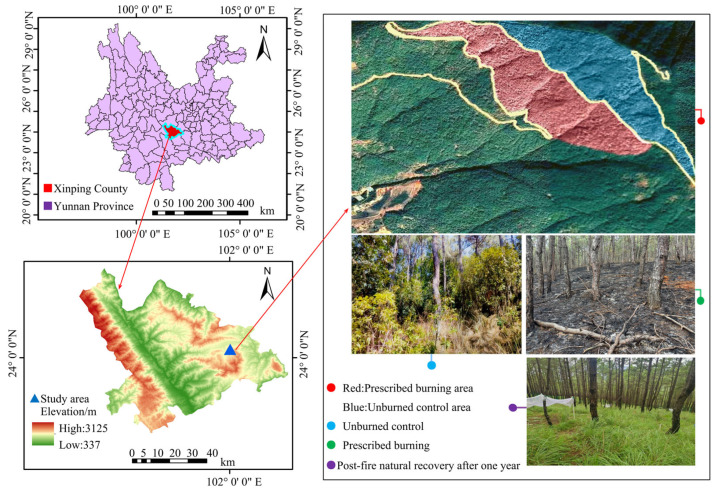
Overview map of the study area.

The study area is located at Zhaobi Mountain in Yuxi City, within the Xinping Yi Autonomous County of Yunnan Province, China (102°0′7″–102°0′8″ E, 24°2′38″–24°2′41″ N), as shown in Figure 1. The region is characterized by mountainous and plateau topography, with a subtropical semi-humid plateau monsoon climate. Elevation ranges from 1990 to 2050 m, displaying a general topographical gradient from lower altitudes in the southeast to higher elevations in the northwest. The dominant soil types are mountainous red soil and basalt-derived red soil, both of which developed from Tertiary ancient red earth and basalt. The climate exhibits marked seasonal variation, with a wet season from May to October and a dry season from November to April. The average annual precipitation is 1050 mm, and the mean annual temperature is 15.1 °C, with recorded extremes of 32 °C and 1 °C. The forest stands, primarily composed of *Pinus yunnanensis*, originated from aerial seeding in the 1980s and have since been maintained through natural thinning and management. The shrub layer is predominantly populated by species such as *Vernonia esculenta*, *Houttuynia cordata*, *Vaccinium bracteatum*, and *Quercus acutissima*. Common herbaceous species include *Elsholtzia ciliata*, *Poa annua*, *Eulalia quadrinervis*, *Filipendula palmata*, and *Viola philippica*. Since 2007, prescribed burning has been implemented annually between late January and mid-February, except in 2020, 2021, and 2024, due to adverse climatic conditions (i.e., excessive drought hindering controlled burns) and concerns over air quality. The ignition strategy for the prescribed burning was downslope fire, with an average flame height of 20 cm (measured with a steel tape measure), an average flame temperature of 510 °C (measured with a handheld infrared thermometer), an average spread rate of 0.2 m/min (measured with stopwatch), and an average blackened height of 1.48 m (measured with a steel tape measure), which is categorized as a low-intensity fire [8,17,30].

The present study, conducted in June 2024, investigates the natural recovery of the soil microbial community 16 months after the 2023 prescribed burns. For this purpose, randomly selected areas of Yunnan pine forest subjected to prescribed burning (PB) and unburned control (UB) were sampled. In each of these forest regions, three sample plots, each measuring 20 m by 20 m, were established, with at least 3 m of distance between adjacent plots. Within each sample plot, three 3 m × 3 m subplots were randomly arranged to further explore the variations within the different treatment areas.

### 2.2. Soil Sampling and Analyses

In each subplot, after removing the understory litter, soil samples were collected from 0–5 cm, 5–10 cm, and 10–20 cm soil layers, using a soil auger in accordance with the five-point sampling method. Debris, including stones and roots, was carefully removed, and the samples were subsequently homogenized to form composite samples for each subplot. The samples were then placed in sterile bags, immediately stored on ice, and transported to the laboratory for analysis. A 2 mm sieve was employed to filter all samples. Some samples were preserved at −80 °C for DNA extraction, while others were stored at 4 °C for ammonium nitrogen (NH_4_^+^-N) measurements. The remaining samples were air-dried at room temperature for subsequent analyses of soil enzyme activity and physicochemical properties.

The cutting ring method was utilized to quantify the bulk density (BD) of the soil [31], and soil moisture (SM) was measured using the gravimetric method after oven-drying [32]. The measurement of soil chemical properties was conducted using the methods outlined by Bao [33]. Soil pH was measured using a pH meter in a 1:5 (*w*/*v*) soil-to-water suspension. Soil organic carbon (SOC) content was checked employing the potassium dichromate-external heating sulfuric acid oxidation method. Soil organic matter (SOM) content was calculated from SOC content using the standard conversion factor (SOM = SOC × 1.724). Soil total nitrogen (TN) content was assessed utilizing the semi-micro Kjeldahl method, while NH_4_^+^-N was quantified through the indophenol blue colorimetric method. Soil total phosphorus (TP), total potassium (TK), and available phosphorus (AP) were determined utilizing inductively coupled plasma optical emission spectrometry (ICP-OES). Available potassium (AK) was determined using flame photometry.

The activities of soil lignin peroxidase (LIP), acid phosphatase (ACP), urease (UE), and sucrase (SC) were determined using enzyme-linked immunosorbent assay (ELISA) kits (Beijing Box Biotechnology Co., Ltd., Beijing, China) [34]. All procedures were conducted strictly in accordance with the manufacturer’s instructions. Enzyme activities were measured spectrophotometrically at wavelengths specified by the kits and calculated based on standard curves. Activities are expressed per gram of soil (U/g), where one unit (U) corresponds to the amount of enzyme required to produce 1 nmol of veratryl alcohol, 1 μg of NH_3_-N, 1 nmol of phenol, or 1 mg of reducing sugar per gram of soil per day.

### 2.3. Soil Microbial DNA Collection and Illumina MiSeq Sequencing

The total DNA of soil microorganisms was extracted using the TIANamp Soil DNA Kit (Tiangen Biotech, Beijing, China). DNA concentrations were quantified using a Nanodrop NC2000 spectrophotometer (Thermo Fisher Scientific, Waltham, MA, USA) to ensure the necessary concentration for PCR amplification. For soil bacteria, PCR amplification of the variable region V3–V4 was implemented utilizing primers 341F (5′-CCTACGGGNGGCWGCAG-3′) and 806R (5′-GGACTACHVGGGTATCTAAT-3′). The reaction mixture consisted of 20 μL, including 10 μL 2 × Phanta Flash Master Mix (Vazyme, Nanjing, China), 2.5 μL ddH_2_O, 2.5 μL forward primer, 2.5 μL reverse primer, and 2.5 μL DNA template. PCR conditions included an initial denaturation at 98 °C for 30 s, followed by 32 cycles (98 °C for 10 s, 57 °C for 5 s, 72 °C for 40 s), and a final extension at 72 °C for 1 min. For soil fungi, the ITS regions were amplified utilizing primers ITS1F (5′-CTTGGTCATTTAGAGAGGAAGTAA-3′) and ITS2R (5′-GCTGCGTTCTTCATCGATGC-3′). The PCR reaction system consisted of 25 μL, including 0.25 μL Q5 High-Fidelity DNA Polymerase (New England Biolabs (NEB), Ipswich, MA, USA), 0.5 μL dNTPs, 5 µL 5 × Q5 High GC Enhancer (New England Biolabs (NEB), Ipswich, MA, USA), 5 µL 5 × Q5 Reaction Buffer (New England Biolabs (NEB), Ipswich, MA, USA), 9.25 μL ddH_2_O, 1.25 μL forward primer, 1.25 μL reverse primer, and 2.5 μL DNA template. The amplification conditions were an initial denaturation at 98 °C for 3 min, followed by 30 cycles (98 °C for 30 s, 55 °C for 30 s, 72 °C for 45 s), and a final extension at 72 °C for 5 min.

Following PCR amplification, library concentration was measured using a microplate reader (BioTek FLx800, Winooski, VT, USA), and product size was verified via 2% agarose gel electrophoresis. The PCR products were pooled at equimolar concentrations and purified with magnetic beads (Vazyme VAHTSTM DNA Clean Beads, Vazyme Biotech Co., Ltd., Nanjing, China). The purified products underwent fluorescence quantification, and the samples were then mixed proportionally to meet sequencing requirements. The TruSeq Nano DNA LT Library Prep Kit (Illumina, Shanghai Personal Biotechnology Co., Ltd., Shanghai, China) was employed to prepare the sequencing library. Library quality was assessed utilizing the Agilent High Sensitivity DNA Kit on an Agilent Bioanalyzer (Agilent Technologies, Inc., Santa Clara, CA, USA). Library DNA concentrations were further quantified employing the Quant-iT PicoGreen dsDNA Assay Kit (Invitrogen, Thermo Fisher Scientific, Waltham, MA, USA) on the Promega QuantiFluor fluorescence quantification system (Promega, Madison, WI, USA). Qualified libraries (with non-repeating index sequences) were gradient-diluted, mixed in corresponding proportions according to the required sequencing volume, denatured into single strands with NaOH, and subjected to paired-end sequencing on the Illumina MiSeq platform (Shanghai Personal Biotechnology Co., Ltd., Shanghai, China).

### 2.4. Bioinformatic Analysis

The Cutadapt plugin in QIIME2 (version 2024.5) was applied to remove primers and unmatched sequences from the raw sequencing data, while the DADA2 (version 1.35.4) plugin was employed for quality control, denoising, merging, and chimera removal. After denoising all libraries, ASV feature sequences and ASV tables were merged, and singleton ASVs (i.e., ASVs with a total read count of 1 across all samples) were removed. High-quality sequences were retained for further analysis. Meanwhile, based on the ITS UNITE database and Silva database (Release 132), fungal and bacterial ASVs were classified and taxonomically annotated using the Naive Bayes classifier in QIIME2.

### 2.5. Statistical Analysis

Microsoft Excel 2016 was adopted to preprocess the data on enzyme activity and soil physicochemical characteristics. GraphPad Prism 10 was used to perform homogeneity of variance (Hartley) and normality (Shapiro–Wilk) tests prior to statistical analysis. The effects of prescribed burning, soil depth, and their interactions on soil physicochemical characteristics, enzyme activity, and α-diversity were evaluated utilizing two-way analysis of variance (ANOVA). For multiple comparisons, Tukey’s test was applied for post hoc multiple comparisons, with significance set at *p* < 0.05. For simple effect tests, the Greenhouse-Geisser adjustment was applied to assess differences across all levels of one component while holding the other factor constant.

Soil microbial α-diversity was evaluated using the Chao1 index (richness) and Shannon index (diversity).

To explore the differences in bacterial and fungal community composition and structure between treatments (PB, UB) and soil depths (SD), Bray–Curtis relative abundance and dissimilarity values were determined based on the ASV × sample matrix. Community composition stacked bar charts, non-metric multidimensional scaling (NMDS), and PERMANOVA analysis were performed utilizing the “ggplot2 (version 3.5.2)” and “vegan (version 2.6-10)” packages in R (version 4.4.2).

Spearman correlation analysis was performed using the “psych (version 2.5.6)”, “reshape2 (version 1.4.4)”, “cowplot (version 1.2.0)”, and “ggplot2 (version 3.5.2)” packages in R (version 4.4.2) to examine the relationship between the relative abundance of dominant taxa and soil physicochemical properties. Distance-based redundancy analysis (RDA) was conducted by applying CANOCO5 to investigate the influence of soil environmental factors on microbial communities. To avoid issues of data overfitting and collinearity, RDA was conducted adopting the following steps: (1) removing explanatory variables with variance inflation factor (VIF) > 10; (2) performing stepwise selection based on Akaike information criterion (AIC) to select the model with the smallest AIC value; (3) conducting significance testing (*p* < 0.05, 9999 permutation tests) on the final model, retaining only significant variables.

Co-occurrence networks of soil bacteria and fungi were built in R (version 4.4.2) to determine key microbial taxa in bacterial and fungal communities and to examine the effects of prescribed burning on these interactions. To simplify the networks, genera having relative abundances less than 0.1% of the bacterial and fungal sequences in each sample were eliminated. Subsequently, a co-occurrence network was constructed based on Spearman correlation coefficients (|r| > 0.8 and *p* < 0.05). Network visualization was performed using Gephi (version 0.10.0), which provided the node scores and pertinent topological metrics. The network was an undirected network with a Fruchterman-Reingold structure, with edges that had no direction. The R (4.4.2) packages “igraph (version 2.1.4)” were employed to calculate network, node, and edge attributes, while the “microeco (version 1.15.0)”, “meconetcomp (version 0.6.1)”, and “ggplot2 (version 3.5.2)” packages were used for analyzing the robustness and topological properties of microbial networks. Meanwhile, the linear fitting analysis between the relative abundance of key microorganisms and the stability of co-occurrence networks was conducted with Origin 2021 software.

## 3. Results

### 3.1. Soil Physicochemical Properties and Enzyme Activities

**Figure 2:**
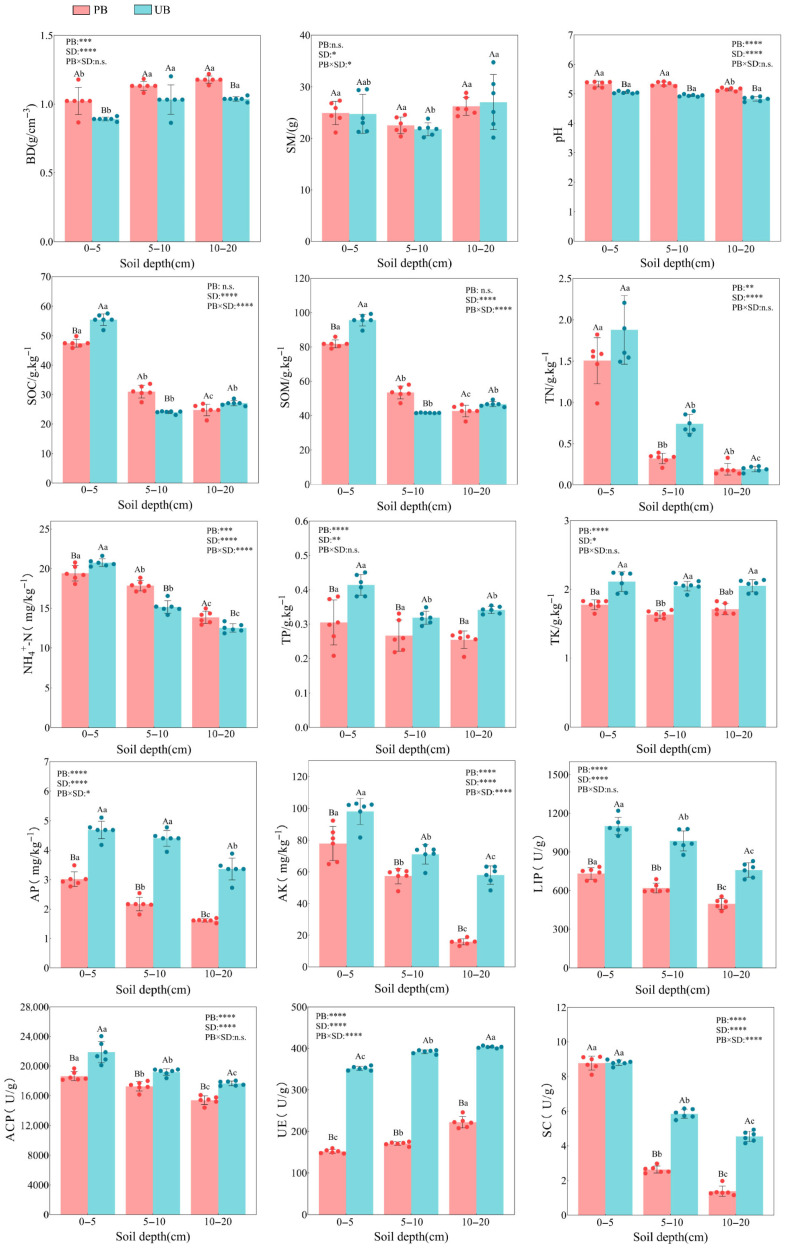
Comparison of soil physicochemical properties and enzyme activities across soil depths (0–5 cm, 5–10 cm, and 10–20 cm) under prescribed burning (PB) and unburned control (UB) treatments. Error bars represent standard deviations. Asterisks denote statistically significant effects of prescribed burning (PB), soil depth (SD), and their interaction (PB × SD): * *p* < 0.05; ** *p* < 0.01; *** *p* < 0.001; **** *p* < 0.0001; ns indicates non-significant effects. Colored dots above the bars represent individual sample values. Different lowercase letters indicate significant differences among soil depths within the same burning treatment (*p* < 0.05), while different uppercase letters denote significant differences between burning treatments within the same soil depth (*p* < 0.05).

As shown in Figure 2, prescribed burning, soil depth and their interaction had significant impacts on soil physicochemical properties and enzyme activities. Compared to the unburned control, prescribed burning significantly increased the BD in the 0–20 cm soil layer (*p* < 0.001), with an overall increase of 9.66–14.73%, where the 0–5 cm layer showed the greatest increase (14.73%). In contrast, SM did not exhibit any significant changes across all soil layers (*p* > 0.05). Soil pH increased significantly by 5.61%, 7.96%, and 6.74% in the 0–5 cm, 5–10 cm, and 10–20 cm layers, respectively (*p* < 0.0001). Prescribed burning had a distinct effect on SOC and SOM, decreasing significantly by 14.44% in the 0–5 cm layer (*p* < 0.0001), increasing by 29.19% in the 5–10 cm layer (*p* < 0.001), and showing no significant changes in the 10–20 cm layer. TN declined by 56.76% (*p* = 0.0015) in the 5–10 cm soil layer, with no significant differences observed in the 0–5 cm and 10–20 cm layers. Conversely, NH_4_^+^-N significantly decreased by 6.47% in the 0–5 cm layer (*p* = 0.018) but increased significantly by 17.73% and 10.66% in the 5–10 cm and 10–20 cm layers, respectively (*p* < 0.01). Furthermore, prescribed burning significantly reduced the total nutrients (TP, TK) and available nutrients (AP, AK) in the 0–20 cm soil depth (*p* < 0.0001), with overall reductions of 16.36–26.40%, 15.92–20.28%, 35.56–52.03%, and 19.32–72.67%, respectively. The reduction in available nutrients was more pronounced than that of total nutrients. For soil enzyme activities, prescribed burning markedly suppressed enzyme activities across the 0–20 cm soil depth (*p* < 0.0001). LIP activity declined by 33.55–37.15%, ACP activity decreased by 9.99–14.77%, and UE activity decreased by 44.94–56.91%. SC showed no significant changes in the 0–5 cm layer but exhibited significant reductions in the 5–10 cm (54.95%) and 10–20 cm (69.53%) layers.

Regarding vertical variations, the majority of soil physicochemical factors, including SOC, SOM, TN, NH_4_^+^-N, AP, AK, LIP, ACP, and SC, exhibited a decreasing trend with increasing soil depth (*p* < 0.05), with particularly significant changes observed between the 0–5 cm and 5–10 cm layers. In contrast, UE activity displayed a significant positive correlation with soil depth (*p* < 0.0001). No vertical differentiation was observed for BD, SM, pH, TK, or TP (*p* > 0.05).

From the perspective of interaction effects, the interaction between prescribed burning and soil depth significantly influenced SM, SOC, SOM, NH_4_^+^-N, AP, AK, SC, and UE (*p* < 0.05), while BD, pH, TN, TP, TK, LIP, and ACP were not significantly affected by the interaction (*p* > 0.05).

### 3.2. Soil Microbial Community Composition

**Figure 3:**
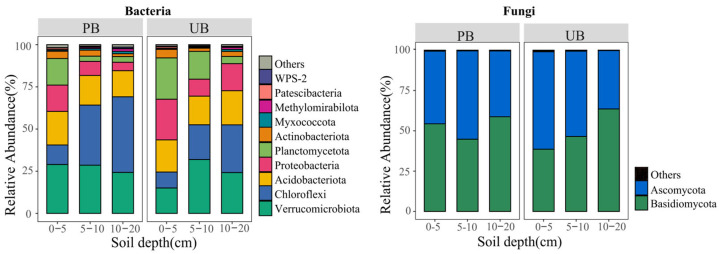
Relative abundance of bacterial and fungal phyla in different soil layers (0–5, 5–10, 10–20 cm) under prescribed burning (PB) and unburned control (UB) conditions.

As shown in Figure 3, prescribed burning considerably increased the number of microbiological phyla across different soil layers. In the PB area, 28, 25, and 19 bacterial phyla, and 14, 11, and 15 fungal phyla were detected in the 0–5 cm, 5–10 cm, and 10–20 cm soil layers, respectively. In contrast, in the UB area, 24, 24, and 20 bacterial phyla and 12, 10, and 10 fungal phyla were detected in the corresponding soil layers, respectively. The bacterial communities were predominantly composed of Verrucomicrobiota (PB/UB: 27.28%/23.73%), Chloroflexi (PB/UB: 30.66%/19.44%), Acidobacteriota (PB/UB: 17.64%/18.75%), and Proteobacteria (PB/UB: 9.66%/16.73%) as the major phyla; whereas the fungal communities were mainly dominated by Basidiomycota (PB/UB: 52.61%/49.55%) and Ascomycota (PB/UB: 46.87%/49.82%). The histogram shows the top 10 bacterial phyla and top 2 fungal phyla with the highest relative abundances in PB and UB, while the remaining taxa are classified as “Others”.

The ANOVA analysis further revealed the effects of prescribed burning on the relative abundances of dominant bacterial phyla across different soil layers (Tables S1 and S2). For bacteria, in the 0–5 cm soil layer, the relative abundance of Verrucomicrobiota increased significantly, while the relative abundances of Chloroflexi, Proteobacteria, and Acidobacteriota did not exhibit significant changes. In the 5–10 cm and 10–20 cm soil layers, the relative abundance of Chloroflexi increased significantly, whereas Proteobacteria decreased significantly, with no significant differences observed in the relative abundances of Verrucomicrobiota and Acidobacteriota. Prescribed burning also had a substantial effect on fungal communities. The relative abundance of Basidiomycota increased significantly in the 0–5 cm soil layer, while that of Ascomycota decreased. No significant changes were observed in fungal phyla in the other soil layers.

Further analysis of dominant bacterial phyla in various soil layers (Table S1) revealed that the relative abundance of Chloroflexi increased with soil depth under both treatments, though the increase between 5–10 cm and 10–20 cm layers was not statistically significant. The relative abundance of Proteobacteria decreased with increasing soil depth in the PB zone, with no significant decrease observed between 5–10 cm and 10–20 cm layers, while no significant vertical gradient pattern was found in the UB zone. Verrucomicrobiota and Acidobacteriota exhibited no significant vertical variation patterns under both treatments. For fungi (Table S1), Basidiomycota and Ascomycota did not show a clear vertical gradient pattern in the PB zone, but in the UB zone, Basidiomycota showed an increasing trend, while Ascomycota exhibited a decreasing trend with soil depth.

The interaction analysis (Table S2) indicated that the Verrucomicrobiota, Chloroflexi, Proteobacteria, Basidiomycota and Ascomycota were all significantly influenced by the interaction between prescribed burning and soil depth. However, the Acidobacteriota was not affected by this interaction.

**Figure 4:**
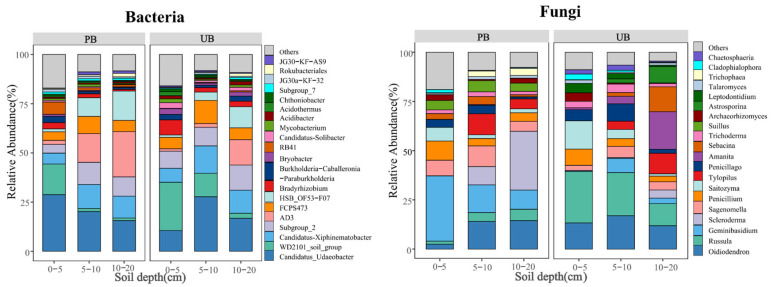
Relative abundance of bacterial and fungal genera in different soil layers (0–5, 5–10, 10–20 cm) under prescribed burning (PB) and unburned control (UB) conditions.

As shown in Figure 4, at the genus level, the PB area identified 291, 202, and 155 bacterial genera in the 0–5 cm, 5–10 cm, and 10–20 cm soil layers, respectively, along with 400, 259, and 215 fungal genera. In contrast, the UB area detected 263, 235, and 190 bacterial genera, and 335, 285, and 238 fungal genera in the corresponding soil layers. These results suggested that prescribed burning enhanced the diversity of bacterial and fungal genera in the 0–5 cm layer, while both PB and UB areas exhibited a decreasing trend in microbial genus abundance with increasing soil depth. The dominant bacterial genera were *Candidatus_Udaeobacter* (PB/UB: 21.53%/18.34%), *WD2101_soil_group* (PB/UB: 6.14%/13.01%), *Candidatus_Xiphinematobacter* (PB/UB: 9.61%/10.89%), and *Subgroup_2* (PB/UB: 8.49%/10.31%). The predominant fungal genera included *Oidiodendron* (PB/UB: 10.33%/14.05%), *Russula* (PB/UB: 3.95%/19.73%), *Geminibasidium* (PB/UB: 18.98%/3.49%), and *Scleroderma* (PB/UB: 13.05%/1.55%). The histogram depicts the top 20 bacterial and fungal genera with the highest relative abundances in PB and UB, with remaining taxa categorized as “Others”.

Further analysis using ANOVA revealed the effects of prescribed burning on the relative abundance of dominant bacterial genera across different soil layers (Tables S1 and S2). For bacteria, in the 0–5 cm soil layer, the relative abundance of *Candidatus_Udaeobacter* increased significantly, while the relative abundance of *Subgroup_2* and *WD2101_soil_group* decreased significantly, with no significant change observed in the relative abundance of *Candidatus_Xiphinematobacter*. In the 5–10 cm and 10–20 cm soil layers, the relative abundance of *WD2101_soil_group* decreased significantly, while no significant differences were observed in the relative abundances of *Candidatus_Udaeobacter* and *Candidatus_Xiphinematobacter*. The relative abundance of *Subgroup_2* did not show a significant difference in the 5–10 cm layer but decreased significantly in the 10–20 cm layer. Similarly, fungal community responses to prescribed burning were also complex (Tables S1 and S2). In the 0–5 cm soil layer, the relative abundance of *Geminibasidium* increased significantly, while that of *Russula* and *Oidiodendron* decreased significantly, with no significant difference observed in the relative abundance of *Scleroderma*. In the 5–10 cm and 10–20 cm soil layers, the relative abundance of *Scleroderma* increased significantly, while *Geminibasidium* showed no significant change. The relative abundances of *Russula* and *Oidiodendron* decreased significantly in the 5–10 cm layer but exhibited no significant difference in the 10–20 cm layer.

Analysis of the dominant genera in different soil layers revealed that (Table S1), for bacteria, the relative abundance of *Candidatus_Udaeobacter* in the PB area decreased with increasing soil depth, though this decrease was not significant. In contrast, the relative abundance of *Candidatus_Udaeobacter* did not show a significant vertical gradient pattern in the UB area. The relative abundance of *WD2101_soil_group* did not exhibit a significant gradient with soil depth in the PB area, while it decreased with increasing soil depth in the UB area. The relative abundances of *Candidatus_Xiphinematobacter* and *Subgroup_2* did not exhibit significant soil layer variation patterns under both treatments. For fungi, the relative abundance of *Scleroderma* increased with soil depth under both treatments, while *Geminibasidium* exhibited a decreasing trend with soil depth increment in the PB area, and did not show a significant soil depth gradient pattern in the UB area. The relative abundance of *Russula* and *Oidiodendron* did not show a significant soil depth change pattern under both treatments.

Interaction analyses indicated (Table S2) that the *Candidatus_Udaeobacter*, *WD2101_soil_group*, *Subgroup_2*, and *Geminibasidium*, *Russula*, *Oidiodendron*, and *Scleroderma* were all significantly influenced by the interaction between prescribed burning and soil depth. However, the *Candidatus_Xiphinematobacter* was not affected by this interaction.

### 3.3. Soil Microbial α-Diversity

**Figure 5:**
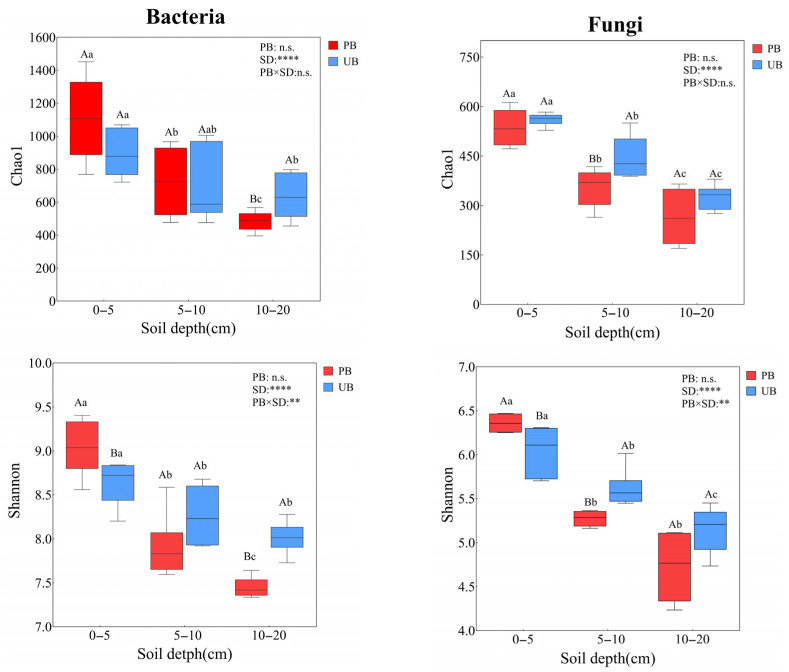
Comparison of microbial α-diversity across soil depths (0–5 cm, 5–10 cm, and 10–20 cm) under prescribed burning (PB) and unburned control (UB) treatments Asterisks indicate statistically significant effects of prescribed burning (PB), soil depth (SD), and their interaction (PB × SD): ** *p* < 0.01; **** *p* < 0.0001; n.s. indicates non-significant effects. Different lowercase letters above boxplots indicate significant differences among soil depths under the same burning treatment (*p* < 0.05), while different uppercase letters denote significant differences between burning treatments within the same soil depth (*p* < 0.05).

As shown in Figure 5, based on the Shannon and Chao1 index analyses, prescribed burning did not have a significant overall effect on the Chao1 and Shannon indices of bacteria and fungi (*p* > 0.05), but it had significant effects on different soil layers. In the 0–5 cm soil layer, prescribed burning did not significantly alter the Chao1 indices of bacterial and fungal communities but increased significantly the Shannon index. In the 5–10 cm soil layer, both the Chao1 and Shannon indices of fungi decreased significantly, whereas no significant differences were observed in bacteria. In the 10–20 cm soil layer, both the bacterial Chao1 and Shannon indices decreased significantly, although no significant differences were detected in fungi. Furthermore, both the Shannon and Chao1 indices for bacteria and fungi exhibited significant vertical stratification patterns (0–5 > 5–10 > 10–20 cm). Interaction analysis revealed a significant interaction effect between prescribed burning and soil depth on the Shannon indices of bacterial and fungal communities (*p* < 0.01), whereas no significant effect was observed on the Chao1 indices (*p* > 0.05).

### 3.4. Soil Microbial β-Diversity

**Figure 6:**
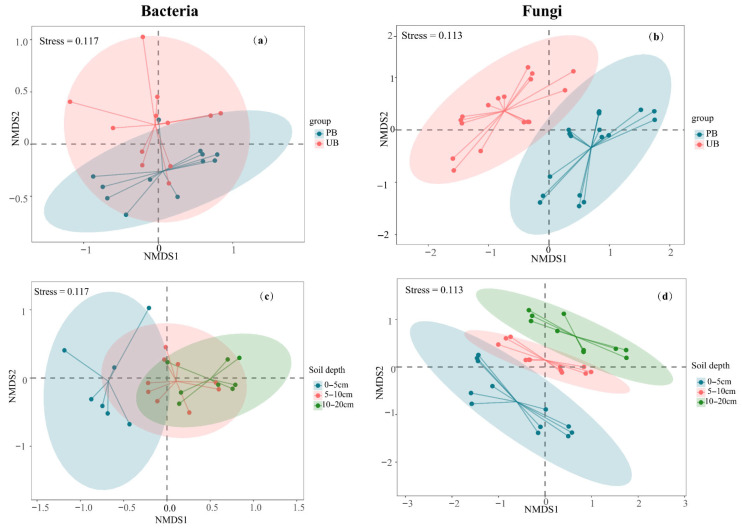
Non-metric multidimensional scaling (NMDS) plots based on the Bray–Curtis dissimilarity, illustrating the effects of prescribed burning on microbial community composition. Panels (**a**) and (**b**) show the overall effects of prescribed burning on bacterial and fungal community structures, respectively, while panels (**c**,**d**) present differences across soil depth layers for bacterial and fungal communities. NMDS1 and NMDS2 represent the two primary ordination axes, with distances between points reflecting relative differences in community composition (unitless). Stress values < 0.2 indicate a reliable representation of the data in reduced dimensions. Points of different colors correspond to distinct treatment or depth groups; shorter distances between points indicate greater similarity in community composition. Ellipses represent 95% confidence intervals for each group.

Prescribed burning significantly altered the structure of the soil microbial community, with varying response patterns between bacteria and fungi, as indicated by the Non-Metric Multidimensional Scaling (NMDS) analysis. Bacterial communities were notably affected by prescribed burning, which also decreased community heterogeneity, and potentially drove community succession (Figure 6a). In contrast, fungal community structure exhibited more distinct separation between PB and UB areas, with more pronounced structural differences than those observed in bacterial communities (Figure 6b). Further analysis revealed vertical stratification characteristics in both the bacterial and fungal communities. The distances between the 0–5 cm layer and both the 5–10 cm and 10–20 cm layers were substantial, demonstrating marked differences in bacterial and fungal community structures between the 0–5 cm layer and other soil layers. In contrast, the community structures of the 5–10 cm and 10–20 cm layers remained relatively similar (Figure 6c,d). These results indicated that the impact of prescribed burning on microbial community structure was predominantly concentrated in the 0–5 cm soil layer. This conclusion was further supported by PERMANOVA analysis (Table S3).

### 3.5. Co-Occurrence Networks of Soil Fungal and Bacterial Communities

**Figure 7:**
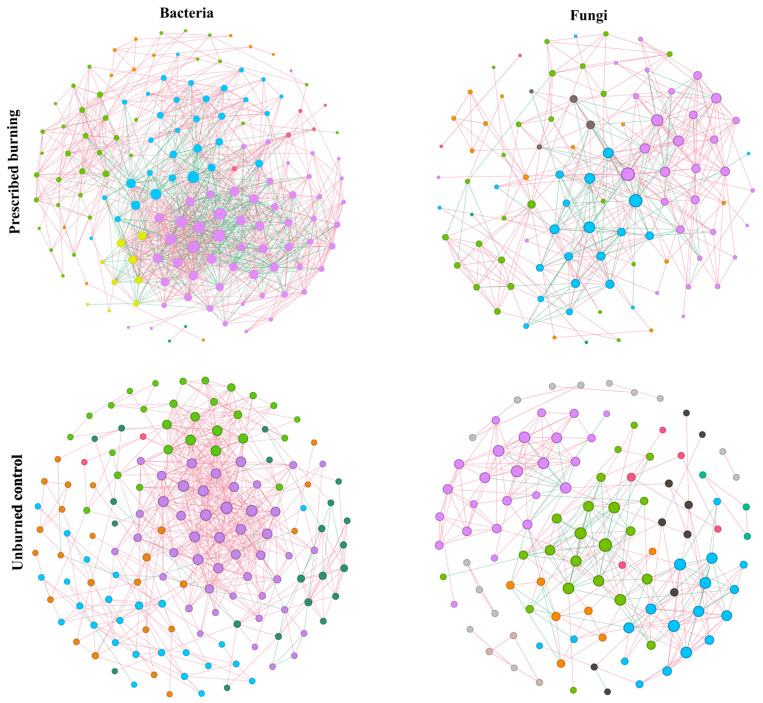
The co-occurrence networks of bacteria and fungi in prescribed burning (PB) and unburned control (UB) plots. In the networks, node size corresponds to the number of connections (degree), while node color represents the modularity class to which each node belongs. Generally, a higher number of submodules indicates lower overall network connectivity. Red edges represent positive (synergistic) associations between nodes, whereas green edges indicate negative (competitive) interactions.

This study demonstrated the symbiotic tendencies within bacterial and fungal communities during the early stages of soil recovery following prescribed burning by constructing bacterial-fungal co-occurrence networks. The results revealed that, compared with the UB area, the bacterial communities in the PB zone exhibited increases of 5 in node count, 520 in edge count, 6.593 in average degree, 0.017 in average clustering coefficient, and 29% in competition ratio. At the same time, decreases of 0.063 in modularity and 29% in cooperation ratio were observed (Table 1). Overall, the topological properties of bacterial networks in the UB area were similar to those in the PB area, with the former demonstrating the highest cooperation ratio (97.38%) (Table 1, Figure 7). The fungal co-occurrence network responded differently to prescribed burning. Fungal networks in the PB area, the number of nodes, modularity, and average clustering coefficient decreased by 9, 0.117, and 0.01, respectively, while the number of edges and average degree increased by 57 and 1.732, with comparable proportions of cooperative and competitive relationships (Table 1). In general, both in the PB and UB areas, bacterial networks were structurally more complex than fungal networks (Figure 7).

**Figure 8:**
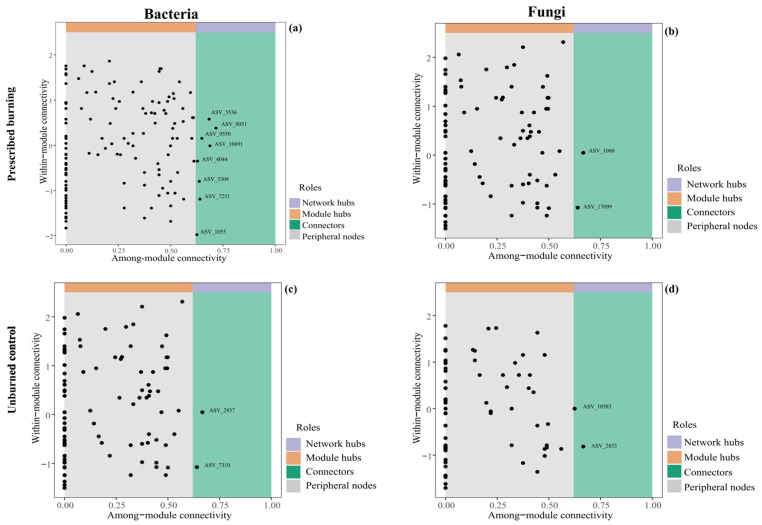
Topological characteristics of bacterial (**a**,**c**) and fungal (**b**,**d**) co-occurrence networks in prescribed burning (PB) and unburned control (UB) treatments. ASV_5536, ASV_10691, and ASV_1055 are affiliated with *Candidatus_Udaeobacter* within the Verrucomicrobiota; ASV_8051 belongs to *Sphingomonas* in the Proteobacteria; ASV_9550 is classified within *AD3* in the Chloroflexi; ASV_4044 represents an unclassified genus within the family *Ktedonobacteraceae* of the Chloroflexi; ASV_3309 is unclassified phylum; ASV_7251 belongs to *TK10* in the Chloroflexi; ASV_1060 and ASV_17099 are also assigned to *Candidatus_Udaeobacter* within the Verrucomicrobiota; ASV_2937 and ASV_7310 belong to unclassified genera in the Ascomycota and unclassified *Aspergillaceae*; ASV_10583 and ASV_2833 are of *Chaetosphaeria* and an unclassified genus, respectively, within the Ascomycota.

Changes in network complexity could result in shifts in the roles of individual taxa within the network. Based on within-module connectivity (Zi) and between-module connectivity (Pi), we identified 8 and 2 bacterial connector nodes in PB and UB areas, respectively (Figure 8a,c), along with an identical number (2) of fungal connectors (Figure 8b,d). These connectors were regarded as keystone microorganisms that play pivotal roles in shaping network structure. In the PB zone, key bacterial taxa primarily belonged to *Candidatus_Udaeobacter* of the Verrucomicrobiota, as well as genera *AD3*, *TK10*, and unclassified genera within the Chloroflexi, and *Sphingomonas* from Proteobacteria. The key fungal taxa were mostly affiliated with the unclassified genera and unclassified *Aspergillaceae* of the Ascomycota. In contrast, the key bacterial taxa in the UB area were *Candidatus_Udaeobacter* from the Verrucomicrobiota, while the key fungal taxa were *Chaetosphaeria* from the Ascomycota and an unclassified phylum. These findings suggested that prescribed burning increased both the diversity and abundance of key bacterial taxa at the phylum and genus levels.

**Figure 9:**
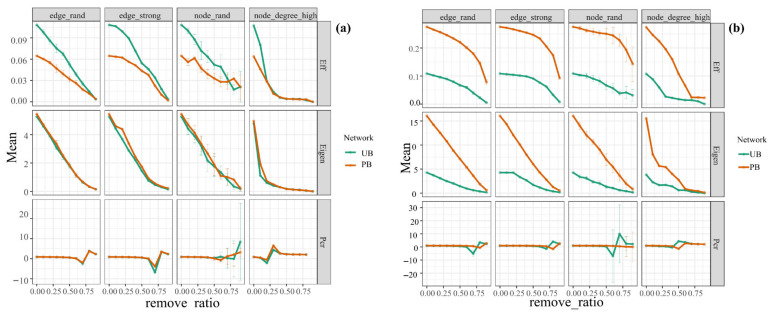
Robustness analysis of microbial co-occurrence networks for bacterial (**a**) and fungal (**b**) communities under prescribed burning (PB) and unburned control (UB) treatments.

We assessed the stability of microbial co-occurrence networks in PB and UB areas by contrasting network efficiency, natural connectivity, and critical removal fractions of vertices (edges) during network disintegration under different removal strategies (edge_rand, edge_strong, node_rand, node_degree_high). The network collapses when a significant portion of its nodes and edges are eliminated above a certain point, known as the percolation threshold. The results demonstrated that the natural connectivity of bacterial communities in the PB area (mean value: 0.95) was slightly higher than that of the UB area (mean value: 0.89), suggesting that the stability of the bacterial community network not only recovered to pre-fire levels after 16 months but also exhibited a rebound in stability. The vertices (edges) critical removal fraction at network dissolution showed no significant difference between PB and UB areas, but the network efficiency was lower than in the UB area, indicating a reduction in synergistic efficiency among populations. Moreover, as the removal rate increased, both the network efficiency and natural connectivity of the bacterial communities in both PB and UB areas declined sharply, demonstrating that the bacterial networks in this region were highly sensitive to both random disturbances and targeted removal of strongly interacting species and keystone species. This suggested the pivotal role of keystone species in maintaining network stability (Figure 9a). Linear regression analysis further confirmed a significant correlation between keystone microorganisms and network stability (R = 0.580, *p* = 0.017) (Figure S1a). Unlike bacterial communities, fungal networks in the PB area generally exhibited higher post-removal network efficiency, natural connectivity, and critical removal fractions of vertices (edges) for network disintegration compared to the UB area. However, their network efficiency and natural connectivity also demonstrated steeper declining trends (Figure 9b). This indicated that their structure may be more vulnerable to disturbances due to lower functional redundancy, structural simplification, and fire-induced homogenization.

### 3.6. Correlation Between Microbial Community Composition and Soil Environmental Factors

**Figure 10:**
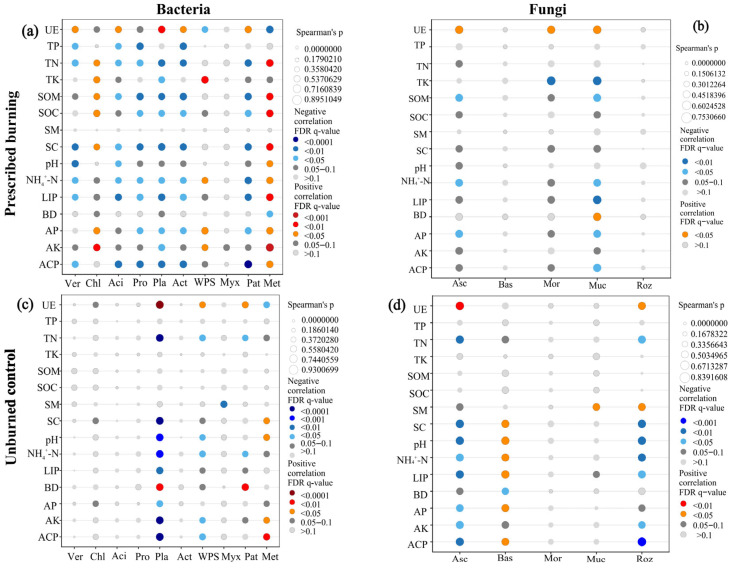
Spearman correlation analysis between the relative abundance of dominant bacterial (**a**,**c**) and fungal (**b**,**d**) communities with soil physicochemical properties in prescribed burning (PB) and unburned control (UB) plots; The color gradient represents Spearman correlation coefficients, with red denoting positive correlations, blue indicating negative correlations, and gray representing no correlation The size of the circles reflects the strength of the correlation, with circles having an outer border indicating positive correlations, while those without an outer border indicating negative correlations. For bacteria, Ver—Verrucomicrobiota; Chl—Chloroflexi; Aci—Acidobacteriota; Pro—Proteobacteria; Pla—Planctomycetota; Act—Actinobacteriota; WPS—WPS-2; Myx—Myxococcota; Pat—Patescibacteria; and Met—Methylomirabilota. For fungi, Asc—Ascomycota; Bas—Basidiomycota; Mor—Mortierellomycota; Muc—Mucoromycota; and Roz—Rozellomycota.

Spearman correlation analysis revealed that prescribed burning significantly altered the association patterns between microbial community composition and soil physicochemical properties. In the PB area, the relative abundance of most bacterial phyla showed significant correlations with soil properties such as UE, TN, SOM, SOC, SC, pH, NH_4_^+^-N, LIP, AP, and ACP. In particular, the bacterial community exhibited positive correlations with UE but negative correlations with most other physicochemical factors (Figure 10a). In contrast, the correlations between the relative abundance of bacterial phyla and soil physicochemical factors were generally weaker in the UB area (Figure 10c). These findings suggested that prescribed burning enhanced the linkage between bacterial community composition and the soil environment. For fungi, prescribed burning significantly diminished the correlations between the relative abundances of Asc, Bas, Roz and soil physicochemical factors, while it strengthened the correlation between the relative abundance of Muc and environmental factors. Specifically, in the PB area, UE showed positive correlations with Asc, Mor, and Muc. BD was positively correlated with Muc, whereas TK, SOM, NH_4_^+^-N, AP, and ACP exhibited negative correlations (Figure 10b,d). Overall, prescribed burning tended to weaken the direct associations between fungal community composition and soil environmental factors.

**Figure 11:**
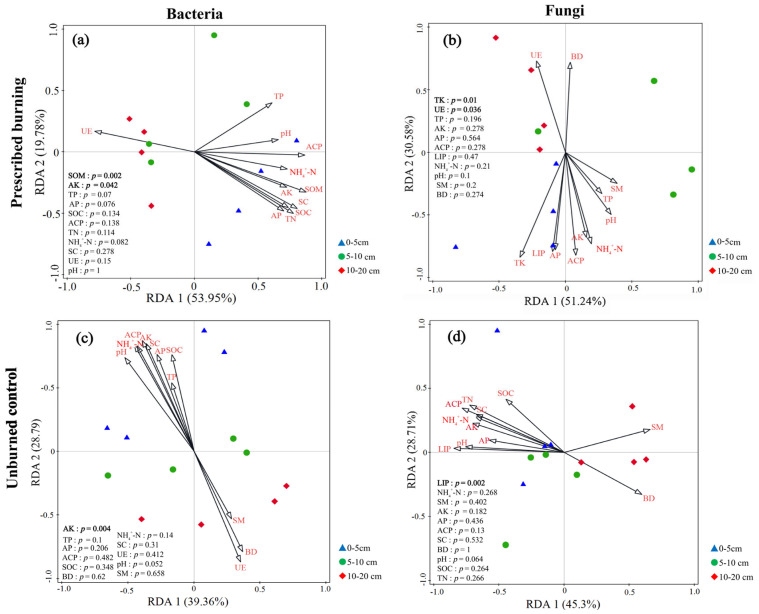
Redundancy (RDA) analysis ordination plots illustrating the relationships between the relative abundance of dominant bacterial (**a**,**c**) and fungal (**b**,**d**) communities and soil physicochemical properties across three soil depths (0–5 cm, 5–10 cm, and 10–20 cm) under prescribed burning (PB) and unburned control (UB) treatments. Different colors and shapes represent distinct sample groups corresponding to treatment and soil depth. Arrows indicate environmental variables; the length of each arrow reflects the strength of the correlation between the environmental factor and microbial community composition, with longer arrows denoting stronger correlations. The angle between an arrow and an RDA axis (RDA1 or RDA2) represents the degree of correlation, where smaller angles suggest stronger associations between the environmental variable and the respective ordination axis.

Redundancy analysis (RDA) was employed to identify the major physicochemical drivers shaping microbial community composition. According to RDA eigenvalues, the first two axes of bacteria in the PB region explained 53.95% and 19.78% of community variation, respectively, accounting for 73.73% of the total variation. Among these, SOM (*p* = 0.002) and AK (*p* = 0.042) were identified as crucial factors influencing bacterial community composition in the PB area (Figure 11a). In contrast, the first two RDA axes for bacteria in the UB region explained 39.36% and 28.79%, respectively, totaling 68.15%, with AK (*p* = 0.004) being the key physicochemical factor regulating community composition (Figure 11c). For fungi, fungal communities in the PB region showed clear separation along RDA axis 1, explaining 51.24% of variation, while the second axis accounted for 30.58%, collectively explaining 81.82% of the total variation. Among these, TK (*p* = 0.01) and UE (*p* = 0.036) were key physicochemical factors regulating the composition of fungal communities in the PB region (Figure 11b). In contrast, the fungi in the UB area showed clear separation along RDA axis 2, which accounted for 28.71% of the variation, while axis 1 explained 45.3%, collectively representing 74.01% of the total variance. LIP (*p* = 0.002) was identified as the key physicochemical factor governing fungal community composition in the UB area (Figure 11d).

## 4. Discussion

### 4.1. The Effects of Prescribed Burning on Soil Physicochemical Properties and Enzyme Activities

This study reveals the complex effects of prescribed burning, soil layer depth, and their interactions on soil physicochemical properties and enzyme activities (Figure 2). Compared with the UB area, prescribed burning significantly increased the BD of the 0–20 cm soil layer, which was consistent with previous research findings [17]. This increase may stem from reductions in surface litter and vegetation biomass after fire, which created exposed patches and facilitated the downward translocation of fine particles during rainfall, thereby occluding soil pores and reducing soil aeration and hydraulic conductivity [35]. Additionally, the ash produced by combustion may play a certain cementing role [36], ultimately leading to increased soil bulk density. It is noteworthy, however, that this study did not observe significant effects of prescribed burning on SM, which was likely attributable to the sampling period occurring during the rainy season when precipitation may have masked potential fire-induced differences in soil moisture. The observed increase in pH across soil layers post-fire may be associated with ash leaching and the release of alkaline cations (e.g., Ca^2+^, Mg^2+^) [37,38], consistent with findings reported in most studies [35,39,40]. SOC and SOM exhibited a complex short-term response pattern, with significant decreases observed in the 0–5 cm soil layer and increases in the 5–10 cm layer post-fire. Similar reductions in carbon concentration and availability after prescribed burns have been reported previously [41,42]. The decrease in surface layers may be attributed to the loss of aboveground plant biomass post-fire, while the increase in deeper SOC and SOM could reflect short-term, localized downward translocation of organic matter mediated by post-fire rainfall leaching. Soil nitrogen was also significantly affected by prescribed burning, with a notable decrease in TN content observed in the 5–10 cm soil layer post-fire. NH_4_^+^-N showed a significant reduction in the 0–5 cm layer but significant increases in the 5–10 cm and 10–20 cm layers, and this increase may be closely related to the decline in Proteobacteria abundance observed in these deeper layers in this study. These findings suggested that the impact of prescribed burning on soil nitrogen was primarily concentrated within the 0–10 cm soil layer, which aligned with observations reported in other nitrogen-limited ecosystems [42,43]. Additionally, prescribed burning significantly reduced the contents of TP, TK, AP, and AK across the 0–20 cm soil layer. This phenomenon may be attributed to the absence of surface vegetation and litter layer post-fire, exposing the soil directly to rainfall erosion and accelerating nutrient loss. Particularly during the sampling period of this study, which coincided with the rainy season featuring abundant precipitation, the leaching and migration of nutrients were further exacerbated. This was especially notable considering that monovalent cations like K^+^ exhibit higher mobility and are more prone to leaching losses [44]. The decline in N and P contents indicated that prescribed burning intensified soil N and P limitations in this *Pinus yunnanensis* forest.

Soil enzyme activity serves as an early and sensitive indicator of soil nutrient status and cycling changes [45]. Prescribed burning significantly reduced the activities of LIP, ACP, and UE in the 0–20 cm soil layer, with these activities decreasing as soil depth increased. This finding was consistent with research conducted in the Jinyun Mountains of Chongqing, China, where the forest is predominantly composed of evergreen broad-leaved forests, mixed coniferous and broad-leaved forests, warm coniferous forests, and bamboo forests [46]. LIP is the primary degrading enzyme involved in lignin biodegradation, and its reduced activity may result from a decrease in microbial diversity associated with LIP secretion due to prescribed burning. ACP plays an essential role in promoting the transformation of soil organic phosphorus and enhancing plant phosphorus uptake efficiency, while UE is vital in soil carbon cycling and the transformation of nitrogen-containing organic matter. The reduction in C, N, and P nutrient contents caused by prescribed burning may be the main reason for the decrease in ACP and UE activities. SC catalyzes the hydrolysis of sucrose into glucose or fructose, providing energy for soil microorganisms. No significant changes in SC activity were observed in the 0–5 cm soil layer after fire, but significant decreases were observed in the 5–10 cm and 10–20 cm layers. This could be related to the impact of prescribed burning on soil pH. The 0–5 cm layer likely maintained SC activity due to increased moisture during the rainy season, which elevated pH values and alleviated soil acidification. The critical role of pH in SC activity was also corroborated by research conducted by Chen et al. [34]. The decrease in SC activity in the 5–10 cm and 10–20 cm soil layers may be attributed to alterations in microbial community composition and the distribution of substrates across different soil layers.

Furthermore, analysis of the soil layer effect revealed that most physicochemical factors, including SOC, SOM, TN, NH_4_^+^-N, AP, AK, LIP, ACP, and SC decreased significantly with increasing soil depth. This trend reflected the vertical stratification pattern of soil nutrients and biological activity, which aligned with findings from numerous studies [13,17].

### 4.2. The Impact of Prescribed Burning on Soil Microbial Communities

This study demonstrated the significant alterations in bacterial and fungal community composition due to prescribed burning (Figure 3). Compared to bacterial genera, fungal genera exhibited more complex and sensitive responses to prescribed burning, supporting our hypotheses (1) and (3). Compared to unburned, prescribed burning significantly increased the relative abundance of Verrucomicrobiota in the 0–5 cm soil layer, which was likely associated with the oligotrophic lifestyle of this phylum [47]. Prescribed burning imposes elevated environmental stress and induces resource limitation, thereby reducing the competitive dominance of many copiotrophic taxa. In contrast, Verrucomicrobiota can persist under nutrient-poor and extreme conditions and exploit complex or recalcitrant organic carbon substrates to sustain growth [48], which likely confers a relative advantage and underlies their increased abundance following burning. Conversely, Proteobacteria showed significant decreases in the 5–10 cm and 10–20 cm soil layers, which may be associated with post-fire ecosystem perturbations and the observed increase in NH_4_^+^-N concentrations in these layers during this study, as well as the significant negative correlation between Proteobacteria and NH_4_^+^-N (Figure 10a). This may be because Proteobacteria includes many denitrifying bacteria that can convert nitrate or nitrite into gaseous nitrogen [49]. The accumulation of NH_4_^+^-N inhibited this process, thereby indirectly affecting the abundance of Proteobacteria. Acidobacteriota did not show significant responses to prescribed burning or soil depth, which was inconsistent with findings from studies indicating reduced Acidobacteriota abundance one year after high-intensity fire disturbance [50]. This discrepancy may be due to differential responses of Acidobacteriota to varying fire intensities, as low-intensity fires might not significantly affect their abundance. The response of the fungal community to prescribed burning differed from that of bacterial communities. In the 0–5 cm soil layer, the relative abundance of Basidiomycota increased, becoming dominant after prescribed burning, while Ascomycota significantly decreased (Figure 3). This finding was in contrast with the conclusion of a study conducted on *Larix gmelinii*, *Pinus koraiensis*, and *Quercus mongolica* [8]. In that northern temperate study area, prescribed burning implemented in November (late autumn) resulted in a significant decrease in the abundance of Basidiomycota and a marked increase in the abundance of Ascomycota. In contrast, our subtropical study area dominated by *Pinus yunnanensis*, experienced prescribed burning in February (spring), with sampling conducted during the summer when precipitation is relatively abundant. Therefore, this discrepancy may be attributed to differences in climate, vegetation, soil nutrient conditions, sampling methods, and prescribed burning timing across study regions, all of which can influence microbial community responses to fire. Soil layer effect analysis revealed that prescribed burning had no significant impact on the vertical distribution patterns of most bacterial phyla, but it significantly weakened the natural gradient distribution of fungal phyla across soil layers. This indicated a greater sensitivity of fungi to prescribed burning compared to bacteria.

However, at the genus level, the bacterium *Candidatus_Udaeobacter* dominated in both PB and UB areas, with prescribed burning significantly increasing its relative abundance in the 0–5 cm soil layer, which aligned with the response of its corresponding phylum to prescribed burning. In contrast, the dominant fungal genera exhibited significant changes: *Geminibasidium* prevailed in the PB area, while *Russula* dominated in the UB area. Prescribed burning notably increased the relative abundance of *Geminibasidium* in the 0–5 cm soil layer, while significantly reducing the abundance of *Russula* in both 0–5 cm and 5–10 cm soil layers (Figure 4). The results indicated differential responses of bacterial and fungal genera to prescribed burning, further confirming that fungi exhibit higher sensitivity to fire than bacteria.

Prescribed burning altered the composition and structure of bacterial and fungal communities but did not change their richness or diversity. This was consistent with findings from a previous study [51]. Moreover, the microbial communities in both PB and UB zones exhibited significant soil layer gradient patterns in richness and diversity (0–5 > 5–10 > 10–20 cm) (Figure 5), which aligned with numerous studies reporting decreased microbial community diversity and abundance with increasing soil depth [9,10,52]. This vertical distribution pattern may be attributed to the gradient effects of environmental factors such as soil organic matter content, nutrient availability, oxygen concentration, and pH, which vary with depth [53,54]. Additionally, prescribed burning significantly altered the soil bacterial and fungal community structures, with more pronounced structural changes observed in fungal communities. The most significant impacts were observed in the 0–5 cm soil layer, while relatively minor effects were detected in deeper soil layers (Figure 6). This result was consistent with the finding that fire disturbance had the most pronounced impact on the microbial community structure in the 0–5 cm layer [13].

Meanwhile, we observed significant changes in the topological properties of microbial networks following prescribed burning (Figure 7). Generally, more complex networks possess more nodes and edges, as well as higher average degrees [55]. The alterations induced by prescribed burning were evident: bacterial communities exhibited increases in nodes, edges, average degree, and average clustering coefficient, suggesting tighter internal connections and the formation of more distinct “clique” structures, thereby enhancing network complexity. In contrast, fungal networks displayed a slight reduction in nodes, along with decreased modularity and internal connection density, although interactions among the remaining species increased marginally. This suggested that fire may have weakened functional specialization within fungal communities, leading to a more homogenized network structure (Figure 7, Table 1). Moreover, complex microbial networks are often indicative of microbial interactions and higher biological activity in the soil [56]. Our findings demonstrated that prescribed burning altered the interactions among microbial taxa. The proportion of competitive relationships among bacteria increased while mutualistic relationships decreased, suggesting that prescribed burning may amplify competition within microbial communities, leading to more negative interactions [57]. This phenomenon may stem from resource limitations, post-fire environmental stress, resource redistribution, and differential adaptability to environmental changes, which could give certain bacterial taxa competitive advantages while leaving others at a disadvantage. For instance, this study found that Verrucomicrobiota (relative abundance: 27.28%) replaced Proteobacteria (relative abundance: 17.64%) and Acidobacteriota (relative abundance: 9.66%) as the dominant phylum (Figure 3), along with the increase in both the quantity (PB/UB: 8/2) and diversity of key bacterial taxa (Figure 8), which aligned with the rise in competitive interactions within the bacterial network. The interactions between fungal communities and the abundance of key fungal taxa showed no significant changes following prescribed burning. Robustness analysis (Figure 9) revealed that bacterial networks under prescribed burning exhibited slightly higher natural connectivity compared to the unburned control, indicating that the stability of bacterial community networks not only recovered to pre-fire levels but also demonstrated a rebound in stability 16 months after prescribed burning. As shown in previous studies, this rebound in network characteristics suggested that microbial communities possess rapid resilience following fire disturbance, enabling swift reconstruction of relatively stable community structures [18]. The vertex (edge) critical removal fractions for network disintegration were comparable, but the network efficiency and natural connectivity plummeted sharply with the removal of nodes and edges, indicating that the key bacterial taxa in this region play a significant role in maintaining network stability (Figure S1a). This aligned with the findings that critical microorganisms are vital for maintaining network stability during ecosystem recovery [20]. For fungi, the natural connectivity, network efficiency, and critical removal fractions at network disintegration were higher in the PB area than in the UB area. As edges and nodes were removed, the fungal network in the UB area exhibited a more gradual decline, suggesting that although prescribed burning enhanced the stability and internal connection efficiency, their lower functional redundancy, simplified structure, and fire-induced homogenization may render them more susceptible to disturbances.

### 4.3. Correlations Between Soil Microbial Community Composition and Environmental Factors

Numerous environmental factors have been identified as key determinants of post-fire changes in soil microbial community composition, including SM, pH, SOC, TN, available nutrients, inorganic nitrogen content, climate change, and aboveground vegetation [8,27,58]. This study demonstrated that prescribed burning reshaped the association patterns between soil microbial community composition and environmental factors. The observed shift in these association patterns highlights the direct impact of prescribed burning on soil nutrient availability. The results showed that bacterial communities in the PB zone exhibited negative correlations with most nutrients (Figure 10a), reflecting their adaptation to nutrient-poor conditions [59,60]. Furthermore, RDA analysis identified SOM and AK content as key factors regulating post-fire soil bacterial community (Figure 11a), which aligned with the findings of another study [27]. Notably, the impact of prescribed burning on fungal communities was markedly different from its effect on bacterial communities. Prescribed burning significantly reduced the correlations between Asc, Bas, and Roz and soil physicochemical factors, while enhancing the correlations between Muc and physicochemical factors (Figure 10b,d). This discrepancy may reflect the diverse ecological strategies employed by different fungal taxa in response to fire disturbance [61]. UE and TK were identified as key factors influencing post-fire soil fungal communities (Figure 11b). The positive correlation between UE activity and fungi provides an explanation for the significant decrease in Asc abundance observed in the 0–5 cm soil layer after fire in this study. Changes in UE activity can affect nitrogen use efficiency and soil nitrogen supply capacity, thereby influencing nitrogen uptake, plant growth, and ultimately the structure and function of microbial communities [62]. Conversely, TK exhibited a negative correlation with fungal communities, a phenomenon also observed in other similar studies [63]. In conclusion, differences in soil nutrient utilization may be one of the reasons for the divergent responses of bacterial and fungal communities in the short term after fire. Therefore, long-term monitoring is required to comprehensively assess the lasting effects of prescribed burning on soil microbial communities and soil nutrient cycling.

## 5. Conclusions

This study observed that prescribed burning enhanced the complexity and stability of bacterial co-occurrence networks, with increases in both the number and diversity of keystone taxa playing a pivotal role in maintaining network stability. Prescribed burning also significantly reduced available and total nutrients along with soil enzyme activities in the 0–20 cm layer, increased BD and pH, and had no significant effect on SM. Most soil physicochemical properties were influenced by the interaction between soil depth and prescribed burning and exhibited decreasing trends with increasing depth. Both bacterial and fungal community compositions were markedly altered, with fungal communities exhibiting more complex responses and higher sensitivity to prescribed burning. Microbial richness and diversity decreased with soil depth but were overall unaffected by prescribed burning. The most pronounced changes in microbial community structure occurred in the 0–5 cm soil layer, with fungal communities being more strongly influenced than bacterial communities. Prescribed burning also reshaped the associations between microbial communities and soil physicochemical factors, enhancing the coupling between bacterial composition and soil properties, which were largely negatively correlated with nutrient availability. Soil organic matter (SOM) and available potassium (AK) were identified as key regulators of post-fire bacterial community composition. In contrast, correlations between fungal communities and physicochemical factors were more complex, with certain fungal taxa showing weakened associations. Urease (UE) and alkaline phosphatase (TK) were identified as critical factors influencing post-fire fungal community composition. These findings provide novel insights into the ecological processes mediated by prescribed burning and offer a scientific basis for optimizing fire management strategies and promoting forest ecosystem recovery. However, this study focused solely on the early post-fire recovery stage and the vertical responses of microbial communities and soil properties, leaving long-term successional dynamics unexplored. Future research should adopt a long-term perspective to comprehensively assess the enduring effects of prescribed burning on soil microbial communities, nutrient cycling, and ecosystem functioning.

## Figures and Tables

**Table 1:** Topological parameters of bacterial and fungal community co-occurrence networks in prescribed burning (PB) and unburned control (UB) treatments.

Network Parameter	PB	UB
Bacteria		
Number of nodes	151	146
Number of edges	1170	650
Average degree	15.497	8.904
Modularity	0.439	0.502
Average clustering coefficient	0.526	0.509
Co-occurrence (%)	0.6838	0.9738
Competition (%)	0.3162	0.0262
Fungi		
Number of nodes	97	106
Number of edges	375	318
Average degree	7.732	6
Modularity	0.544	0.661
Average clustering coefficient	0.617	0.627
Co-occurrence (%)	0.7493	0.7484
Competition (%)	0.2507	0.2516

## Data Availability

The original contributions presented in this study are included in the article/Supplementary Material. Further inquiries can be directed to the corresponding author.

## Supplementary Materials

The following supporting information can be downloaded at: https://www.mdpi.com/article/10.3390/microorganisms13092070/s1, Figure S1: Linear regression fitting between key microbial taxa and network stability of bacteria (a) and fungi (b); Table S1: Analysis of variance (ANOVA) of bacterial and fungal phyla and genera across soil depths (0–5 cm, 5–10 cm, and 10–20 cm) under prescribed burning (PB) and unburned control (UB) treatments; Table S2: Two-way analysis of variance (ANOVA) assessing the effects of prescribed burning, soil depth, and their interaction on the relative abundances of dominant bacterial and fungal phyla and genera; Table S3: Permutational multivariate analysis of variance (PERMANOVA) assessing the effects of prescribed burning (PB), soil depth (SD), and their interaction (PB × SD) on bacterial and fungal community dissimilarities.
