# Supplementary material for: Prescribed Burning Enhances the Stability of Soil Bacterial Co-Occurrence Networks in Pinus yunnanensis Forests in Central Yunnan Province, China
Source: Microorganisms. 2025 Sep 5;13(9):2070. doi: 10.3390/microorganisms13092070 (PMC12472813; doi:10.3390/microorganisms13092070)
Supplement: Supplementary file 1 [file microorganisms-13-02070-s001.zip › microorganisms-3798756-supplementary.pdf]

Table S1 Analysis of variance (ANOVA) of bacterial and fungal phyla and genera across soil depths (0–5 cm, 5–10 cm, and 10–20 cm) under prescribed burning (PB) and unburned control (UB) treatments. Different lowercase letters indicate significant differences among soil depths within the same burning treatment ($p < 0.05$), while different uppercase letters indicate significant differences between burning treatments within the same soil depth ($p < 0.05$). Values are presented as means (± standard error).

	PB			UB		
	0 - 5	5 - 10	10 - 20	0 - 5	5 - 10	10 - 20
**Bacteria (Phylum）**						
Verrucomicrobiota	0.2779(0.03899)Aa	0.2811(0.01693)Aa	0.2404(0.04457)Aa	0.1689(0.01616)Bb	0.3527(0.0112)Aa	0.2088(0.02146)Ab
Chloroflexi	0.1155(0.05217)Ab	0.3493(0.02298)Aa	0.4371(0.03273)Aa	0.1066(0.03223)Ab	0.2176(0.03134)Ba	0.2736(0.0313)Ba
Acidobacteriota	0.1984(0.02172)Aa	0.1728(0.008179)Aa	0.1542(0.02775)Aa	0.1976(0.02157)Aa	0.1883(0.03008)Aa	0.1958(0.0471)Aa
Proteobacteria	0.1922(0.01582)Aa	0.0646(0.01231)Bb	0.0573(0.01141)Bb	0.2017(0.03327)Aa	0.1258(0.03406)Aa	0.1950(0.02529)Aa
Planctomycetota	0.1571(0.02161)Ba	0.0303(0.02137)Bb	0.0321(0.00203)Ab	0.3467(0.06515)Aa	0.199(0.052)Ab	0.0413(0.02578)Ac
**Fungus (Phylum）**						
Basidiomycota	0.5433(0.01666)Aa	0.4477(0.01337)Ab	0.5872(0.0183)Aa	0.3859(0.01515)Bc	0.4651(0.02215)Ab	0.6396(0.02868)Aa
Ascomycota	0.4504(0.01593)Ab	0.5478(0.01325)Aa	0.4079(0.01825)Ab	0.604(0.01391)Ba	0.5285(0.02131)Ab	0.3576(0.02779)Ac

**Bacteria (Genus）**						
Candidatus_Udaeobacter	0.2880(0.02914)Aa	0.2019(0.03449)Aa	0.1559(0.02855)Aab	0.1233(0.01875)Bb	0.2956(0.04163)Aa	0.1874(0.03353)Aa
WD2101_soil_group	0.1554(0.02737)Ba	0.01789(0.02613)Bb	0.01338(0.00148)Bb	0.3835(0.1097)Aa	0.178(0.1024)Ab	0.02563(0.023)Ac
Candidatus_Xiphinematobacter	0.0550(0.02183)Ab	0.1220(0.01216)Aa	0.1110(0.01469)Aa	0.0835(0.01817)Ab	0.1477(0.01768)Aa	0.1259(0.00088)Aa
Subgroup_2	0.0444(0.01428)Bb	0.1227(0.00229)Aa	0.0977(0.01651)Ba	0.1020(0.00488)Aa	0.1131(0.01672)Aa	0.1542(0.02088)Aa
AD3	0.0205(0.01654)Ab	0.171(0.01739)Aa	0.2299(0.02912)Aa	0.0179(0.00211)Ab	0.0227(0.02882)Bb	0.1237(0.02807)Ba
HSB_OF53-F07	0.0149(0.02705)Ab	0.0946(0.01438)Aa	0.1488(0.01492)Aa	0.0139(0.00592)Ac	0.0511(0.00263)Ab	0.1137(0.00745)Ba
Bradyrhizobium	0.0316(0.01405)Aa	0.0200(0.0071)Aa	0.0084(0.00763)Ba	0.0906(0.03655)Aa	0.0267(0.03463)Aa	0.0312(0.00567)Aa
Burkholderia-Caballeronia-Paraburkholderia	0.0318(0.01004)Aa	0.0118(0.00934)Aa	0.0099 (0.00184)Aa	0.0317(0.0115)Aa	0.0161(0.01341)Aa	0.0288(0.005414)Aa
Bryobacter	0.0105(0.00502)Aa	0.0055(0.00678)Aa	0.0037(0.001759)Ba	0.0342(0.01735)Aa	0.0169(0.01066)Aa	0.0235(0.006691)Aa
RB41	0.0624(0.01023)Aa	0.0129(0.00833)Ab	0.0105(0.00338)Ab	0.0021(0.000162)Bb	0.0043(0.00178)Bb	0.0147(0.001761)Aa
**Fungi (Genus）**						

Geminibasidium	0.2738(0.04716)Aa	0.0732(0.07006)Ab	0.0443(0.02376)Ab	0.0031(0.01593)Bb	0.0620 (0.00665)Aa	0.0155(0.01953)Aab
Russula	0.0131(0.00478)Ba	0.0269(0.00806)Ba	0.0307(0.00682)Aa	0.1661(0.0243)Aa	0.1399(0.02082)Aa	0.0714(0.02229)Aab
Oidiodendron	0.0205(0.00366)Bc	0.0786(0.00434)Ba	0.0554(0.00621)Ab	0.0823(0.01008)Aa	0.1091(0.01458)Aa	0.0721(0.01049)Aab
Sagenomella	0.0663(0.0127)Aa	0.0584(0.01336)Aa	0.0201(0.00424)Ab	0.0170(0.003232)Bb	0.0359(0.00331)Ba	0.0246(0.00401)Aab
Scleroderma	0.0003(0.0176)Ac	0.0459(0.01467)Ab	0.1191(0.03153)Aa	0.0001(0.00147)Ac	0.0051 (0.0139)Bb	0.0229(0.01469)Ba
Penicillium	0.0808(0.06025)Aa	0.0206(0.06487)Ab	0.0159(0.00462)Ab	0.0537(0.0281)Aa	0.0256(0.03718)Aa	0.0165(0.00908)Aa
Saitozyma	0.0574(0.00605)Aa	0.0107(0.00431)Aab	0.0076(0.0025)Ab	0.0807(0.0068)Aa	0.0306(0.00915)Ab	0.0067(0.00678)Ac
Penicillago	0.0330(0.00544)Aa	0.0257(0.00949)Aa	0.0044(0.0068)Bb	0.0320(0.01444)Aa	0.0586(0.00423)Aa	0.0144(0.01744)Aa

Table S2 Two-way analysis of variance (ANOVA) assessing the effects of prescribed burning, soil depth, and their interaction on the relative abundances of dominant bacterial and fungal phyla and genera. *$p < 0.05$, **$p < 0.01$, ***$p < 0.001$, **$p < 0.0001$; ns indicates no significant difference.

	Prescribed burning	Soil depth	Prescribed burning×depth
**Bacteria（Phylum）**			
Verrucomicrobiota	ns	$p < 0.01$	$p < 0.01$
Chloroflexi	$p < 0.0001$	$p < 0.0001$	$p < 0.05$
Acidobacteriota	$p > 0.05$	$p > 0.05$	$p > 0.05$
Proteobacteria	$p < 0.05$	$p < 0.01$	$p < 0.05$
Planctomycetota	$p < 0.001$	$p < 0.001$	$p < 0.05$
**Fungus（Phylum）**			
Basidiomycota	ns	$p < 0.0001$	$p < 0.0001$
Ascomycota	ns	$p < 0.0001$	$p < 0.0001$
**Bacteria（Genus）**			
Candidatus_Udaeobacter	ns	$p < 0.05$	$p < 0.001$
WD2101_soil_group	$p < 0.01$	$p < 0.0001$	$p < 0.01$
Candidatus_Xiphinematobacter	$p < 0.01$	$p < 0.01$	ns
Subgroup_2	$p < 0.05$	$p < 0.01$	$p < 0.01$
AD3	$p < 0.01$	$p < 0.0001$	$p < 0.01$
HSB_OF53-F07	$p < 0.05$	$p < 0.0001$	ns
Bradyrhizobium	$p < 0.05$	ns	ns
Burkholderia-Caballeronia-Paraburkholderia	ns	ns	ns
Bryobacter	$p < 0.05$	ns	ns
RB41	$p < 0.01$	$p < 0.01$	$p < 0.0001$
**Fungi（Genus）**			

Geminibasidium	$p < 0.001$	$p < 0.05$	$p < 0.001$
Russula	$p < 0.001$	$p < 0.01$	$p < 0.001$
Oidiodendron	$p < 0.001$	$p < 0.0001$	$p < 0.01$
Sagenomella	$p < 0.05$	$p < 0.01$	$p < 0.001$
Scleroderma	$p < 0.0001$	$p < 0.01$	$p < 0.01$
Penicillium	ns	$p < 0.0001$	$p < 0.05$
Saitozyma	$p < 0.05$	$p < 0.0001$	$p < 0.05$
Penicillago	ns	ns	ns

Table S3 Permutational multivariate analysis of variance (PERMANOVA) assessing the effects of prescribed burning (PB), soil depth (SD), and their interaction (PB × SD) on bacterial and fungal community dissimilarities. Statistically significant effects ($p < 0.05$) are indicated in bold.

Factor	Df	Bacteria			Fungi		
		F statistic	R^2 value	*P* value	F statistic	R^2 value	*P* value
Prescribed burning	1	2.6693	0.9647	**0.004**	9.6046	0.2254	**0.001**
Soil depth	2	3.5680	0.2292	**0.001**	4.3278	0.2129	**0.001**
Prescribed burning×depth	5	3.2652	0.4374	**0.001**	7.1012	0.5504	**0.001**

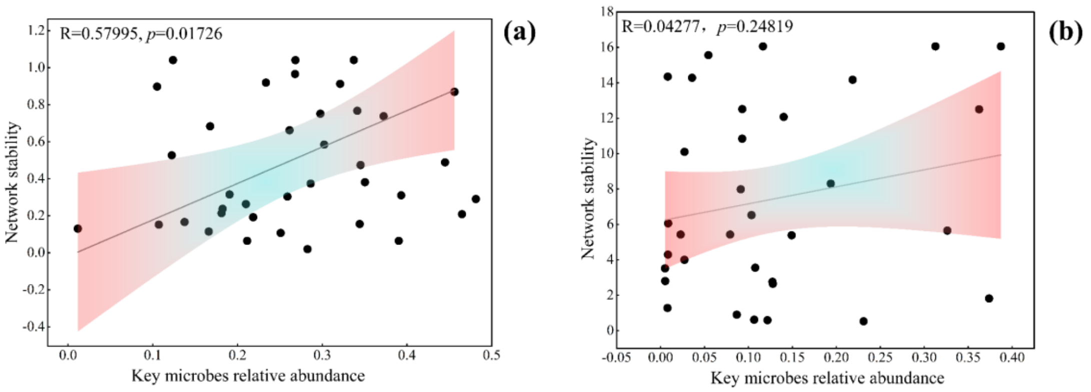

Figure S1. Linear regression fitting between key microbial taxa and network stability of bacteria (a) and fungi (b). Shaded areas represent 95% confidence intervals.
